# Subregional localization and characterization of Ly6aGFP-expressing hematopoietic cells in the mouse embryonic head

**DOI:** 10.1016/j.ydbio.2016.05.031

**Published:** 2016-08-01

**Authors:** Zhuan Li, Chris S. Vink, Samanta A. Mariani, Elaine Dzierzak

**Affiliations:** a University of Edinburgh, Centre for Inflammation Research, Queens Medical Research Institute, Edinburgh, UK; b Erasmus MC Stem Cell Institute, Departments of Cell Biology and Genetics, Erasmus Medical Center, Rotterdam, The Netherlands

**Keywords:** Hematopoietic stem cells, Embryonic head, Ly6aGFP, EHT, Hematopoietic clusters, Hemogenic endothelium

## Abstract

Hematopoietic cell generation in the midgestation mouse embryo occurs through the natural transdifferentiation of temporally and spatially restricted set of hemogenic endothelial cells. These cells take on hematopoietic fate in the aorta, vitelline and umbilical arteries and appear as hematopoietic cell clusters that emerge from the vascular wall. Genetic and live imaging data have supported this. Recently, the embryonic head has been shown to contain fully functional hematopoietic stem cells (HSC). By lineage tracing, cerebrovascular specific endothelial cells were shown to contribute to the postnatal mouse hematopoietic system. Since Ly6aGFP is a marker of all HSCs, some hematopoietic cluster cells and hemogenic endothelial cells in the midgestation mouse aorta, we examine here whether embryonic head HSCs and vascular endothelial cells are positive for this marker. Whereas some head vasculature, single hematopoietic cells and all HSCs are Ly6aGFP expressing, we do not find clusters of hematopoietic cells emerging from the cerebrovasculature that are characteristic of endothelial-to-hematopoietic transition.

## Highlights

• All midgestation mouse head HSCs and some HPCs are Ly6aGFP expressing.
• Ly6aGFP is expressed in the major head vasculature, particularly in the hindbrain.
• No hematopoietic cell clusters are found within the lumen of the head vasculature.

## 1 Introduction

The generation of the hematopoietic cells by vascular endothelial cells in the mouse embryo has been demonstrated by lineage tracing (Chen et al., 2009, Zovein et al., 2008) and 3-dimensional static/live imaging studies of the embryonic aorta (Bertrand et al., 2010, Boisset et al., 2010, Kissa and Herbomel, 2010). Clusters of hematopoietic cells along the midgestation dorsal aorta and vitelline and umbilical arteries have been phenotypically characterized, imaged *in situ* and show a transition from the expression of endothelial markers such as VE-cadherin, Flk1 and Tie2 in the cells underlying the clusters, to the expression of hematopoietic markers CD41, ckit, CD45 and others in cluster cells (Robin et al., 2011, Rybtsov et al., 2011, Yokomizo and Dzierzak, 2010, Yokomizo et al., 2011). All cluster cells along these arteries express ckit and quantitative analyses show that the number of clusters peaks to about 650 at E10.5, when HSCs are first detected (Yokomizo and Dzierzak, 2010). Functional assays of sorted AGM/vitelline/umbilical artery cells demonstrate that hematopoietic stem cells (HSC) and hematopoietic progenitor cells (HPC) express ckit, CD41, CD45, Runx1 and CD31 (Dzierzak and Speck, 2008, North et al., 2002, Robin et al., 2011, Yokomizo and Dzierzak, 2010). Importantly, the Ly6aGFP marker defines all HSCs in the mouse midgestation AGM, aorta/vitelline/umbilical arteries and placenta, some cluster cells and underlying ventral aortic endothelial cells (de Bruijn et al., 2002, Ottersbach and Dzierzak, 2005) and time lapse imaging of the embryonic aorta shows that the Ly6aGFP expressing endothelial cells undergo endothelial-to-hematopoietic transition (EHT) (Solaimani Kartalaei et al., 2015). Other highly vascular tissues such as the yolk sac, placenta and embryonic head also generate hematopoietic cells (Li et al., 2012, Rhodes et al., 2008; Lux et al., 2008). Recently it has been shown that EHT occurs in the yolk sac to give rise to hematopoietic progenitor cells (Frame et al., 2016). Here we examine the head and the head vasculature of Ly6aGFP embryos for hematopoietic cells, HPC and HSC function and show that Ly6aGFP expression marks some vascular endothelial and hematopoietic cells and all HSCs, but find little evidence of multicellular hematopoietic cluster formation or characteristic of EHT.

## 2 Methods and materials

### 2.1 Mouse and embryo production

*Ly5.1* female (6–8 week) mice and C57BL/6 mice were obtained (Charles River, Harlan). *Ly6aGFP* mice were maintained as hemizygotes on a C57BL/6 background, and transgenic embryos were phenotyped by tail GFP fluorescence. Day of plugging was considered as embryonic day (E) 0. E10.5 corresponds to embryos with 34–40 somite pairs (sp); E11.5 with >40 sp; E12.5 by eye pigmentation and limb webbing. Dissections and cell preparation were done as previously described (Medvinsky et al., 2008). The cell numbers at E10.5 for whole head were 7.8±3.4×10^5^, for forebrain (FB) 2.3±0.6×10^5^, for mid-brain (MB) 1.0±0.5×10^5^, for hindbrain and branchial arches (HBA) 3.2±1.3×10^5^ and at E11.5 for whole head 4.8±9.1×10^6^, for FB 1.5±7.3×10^6^, for MB 4.4±2.9×10^5^, for HBA 1.9±1.0×10^6^. At E12.5 whole head contained 9.9±1.3×10^6^ cells. All animal procedures were approved under UK Home Office regulations and performed in compliance with Standards for Care and Use of Laboratory Animals.

### 2.2 Hematopoietic progenitor and stem cell assays

Clonogenic analysis was performed on sorted cells plated in methylcellulose (M3434; StemCell Technologies). Hematopoietic colonies were counted at day 6 and 12. HSC activity of sorted or unsorted *Ly5.2* head cells (various cell doses) was analysed by *in vivo* transplantation. Cells were intravenously coinjected with 2×10^5^ *Ly5.1* spleen cells into irradiated (9Gy split-dose, γ irradiation) *Ly5.1* recipients. After 16 weeks, donor chimerism (CD45.2) was analysed by flow cytometric analysis on blood after erythrocyte lysis (Beckman Coulter) and antibody staining (7-amino-actinomycin D or Hoechst staining for viability). Multilineage donor chimerism was analysed in recipient blood, bone marrow, spleen, lymph node and thymus with antibodies specific for macrophages (CD11b), granulocytes (Gr1), B (CD19) and T (CD3, CD4, CD8) lymphocytes and erythroid cells (Ter119). For secondary transplantations, BM cells (3×10^6^) cells from primary recipients were injected into irradiated *Ly5.1* recipients.

### 2.3 Immunostaining

Immunostaining was performed as previously described (Ling et al., 2004). E10.5, E11.5 and E12.5 *Ly6aGFP* embryos were fixed (2% paraformaldehyde/PBS, 4 °C, 1 h for E10 head, 2 h for E11.5 head and 2.5 h for E12 head). Embryonic heads were equilibrated in 20% sucrose/PBS at 4 °C overnight and then embed in the Tissue Tek before freezing. 10-μm cryosections were prepared. Endogenous biotin activity was blocked by Avidin/Biotin blocking kit. The fixed head sections were incubated with primary antibodies (ckit (2B8), GFP, Runx1 (EPR3099)) or secondary antibodies (Anti-Rabbit Alexa Fluor® 488 IgG (H+L), anti-rat Alexa Flour 555 IgG(H+L), Anti-Rabbit Alexa Fluor® 647 IgG (H+L)(1−2) into PBS-block (PBS containing 0.05% tween and 1% BSA) overnight and washed three times in PBS-T (PBS with 0.05% tween). Samples were stained with DAPI for 10 min, room temperature and then mounted with mounting buffer. Images were acquired with an inverted confocal microscope (Leica SP5) and processed using Leica AF Lite.

## 3 Results and discussion

### 3.1 Embryonic head contains Ly6aGFP expressing hematopoietic cells

**Fig. 1:**
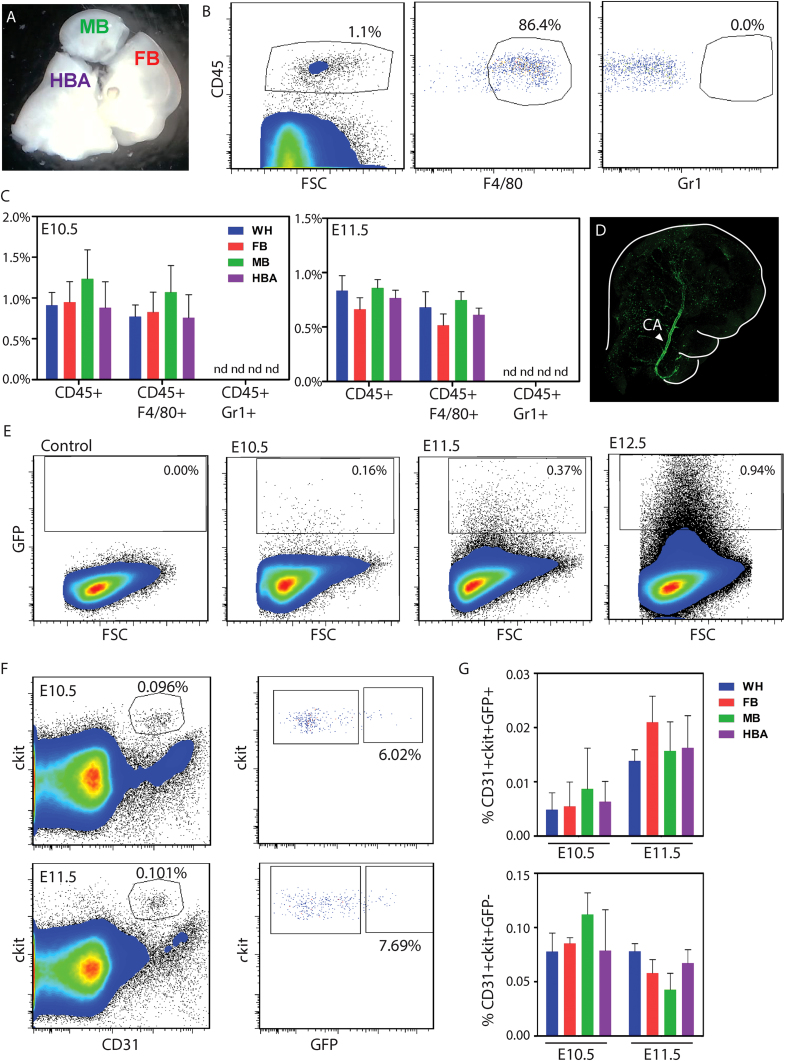
Localization of phenotypic hematopoietic and Ly6aGFP expressing cells in the embryonic head. (A) Subregional dissection of an E11.5 mouse head into the forebrain (FB), midbrain (MB) and hindbrain with brachial arches (HBA). Number of cells x 1000 per E10.5 and E11.5 whole head=78.2±34.7 and 484±91.7; FB=23.9±6.3 and 154.0±73.4; MB=10.0±4.8 and 43.79±29.0; HBA=32.2±13.4 and 190.7±101.2 respectively. (B) Representative flow cytometric cell density plots for E10.5 whole head single cell suspensions. Viable CD45 positive cells (against forward scatter (FSC)) are gated and analysed for F4/80 and Gr1 expression. (C) Bar graph showing percentages of CD45^+^, CD45^+^F4/80^+^ and CD45^+^Gr1^+^ cells are shown for whole head (WH, blue), forebrain (FB, red), midbrain (MB, green) and hindbrain with brachial arches (HBA, purple). n=3. Mean±SD. nd=not detected. (D) Confocal image of whole mount E11.5 *Ly6aGFP* head (right halfsection) showing GFP expression (green fluorescence) in the carotid artery (CA, arrowhead). An outline around the boundaries of the head is drawn to facilitate orientation. (E) Representative flow cytometric cell density plots for non-transgenic and *Ly6aGFP* E10.5, E11.5 and E12.5 whole head single cell suspensions. Percentages of GFP^+^ cells are shown. n=3. (F) Representative flow cytometric density plots showing percentages of ckit^+^CD31^+^ cells and GFP^+^ cells in the gate indicated. (G) Bar graph showing the percentage of ckit^+^CD31^+^GFP^+^ and ckit^+^CD31^+^GFP^−^ cells in E10.5 and E11.5 whole head (blue) and head subregions FB (red), MB (green) and HBA (purple). n=3 Mean±SD.

Since the head is composed of large numbers of non-hematopoietic cells, we attempted to localize head hematopoietic cells by subdissection (Fig. 1A) according to developmentally defined regions – forebrain (FB), midbrain (MB) and hindbrain (with attached brachial arches: HBA). In addition, we used the *Ly6aGFP* transgenic mouse model (de Bruijn et al., 2002, Ma et al., 2002) as a localization and potential enrichment marker for head hemogenic endothelium and HSCs. Analysis of the whole E11.5 head and the three head subregions showed large numbers of CD45^+^ and F4/80^+^ cells. The percentages of these cells was similar between the 3 subregions (Fig. 1B and C) and are likely to be yolk sac tissue resident macrophages that are dispersed throughout the brain as others have reported (Ginhoux et al., 2010, Gomez Perdiguero et al., 2015). No Gr1^+^ cells were detected.

To more specifically examine the hematopoietic and endothelial compartments, *Ly6aGFP* transgenic embryos were examined. Whole head confocal imaging (Fig. 1D) showed the strongest GFP expression along some areas of the major head vasculature, such as the rostral extension of the dorsal aorta (carotid artery (arrow)) and single GFP^+^ cells scattered throughout the head. At E10.5, E11.5 and E12.5 flow cytometric analyses detected 0.20±0.04%, 0.39±0.03% and 0.97±0.16% GFP^+^ cells respectively (n=3) in whole head cell suspensions (Fig. 1E). Other markers such as cKit and/or CD31 that characterize aortic hematopoietic cluster cells and endothelial cells were found by flow cytometric analysis to be expressed by some cells of the embryonic head. At E10.5 1.26±0.42% and E11.5 0.52±0.07% of head cells were ckit^+^, and 1.89±0.81% of E10 and 1.03±0.13% of E11 head cells were CD31^+^ (n=3). Interestingly, the frequency of CD31^+^cKit^+^GFP^+^ cells (phenotypic HSCs) increased between E10.5 and E11.5 (Fig. 1F and G). These frequencies were not different between the subregions indicating that the phenotypic hematopoietic cells were distributed throughout the embryonic head.

### 3.2 HPCs in the embryonic head are predominantly Ly6aGFP negative

**Fig. 2:**
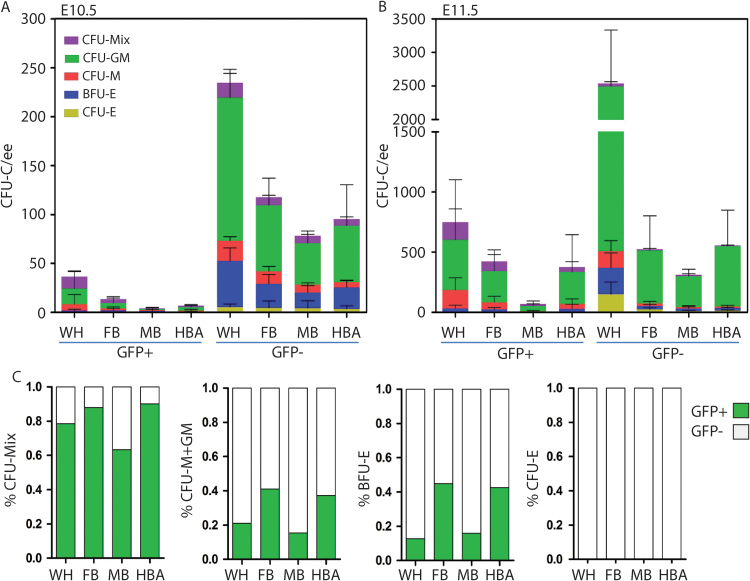
Head hematopoietic progenitors are predominantly Ly6aGFP negative. (A) E10.5 and (B) E11.5 *Ly6aGFP* head single cell suspensions were sorted for GFP^+^ and GFP^−^ cells and plated in methylcellulose. Colony numbers and types per embryo equivalent (ee) of whole head (WH), forebrain (FB), midbrain (MB) and hindbrain with brachial arches (HBA) were counted on day 4 or day 12. CFU (colony forming unit) -Mix (granulocyte, erythrocyte, megakaryocyte, macrophage), -GM (granulocyte, macrophage), -M (macrophage) and -E (erythrocyte) and BFU-E (burst forming unit-erythrocyte) are shown. n=3. Mean±SD. (C) Bar graphs showing the percentage of each colony type in the GFP^+^ (green) and GFP^−^ (white) sorted fractions of WH, FB, MB and HBA for E11.5 *Ly6aGFP* embryos.

Ly6aGFP is a distinguishing marker of the most immature hematopoietic cells as they are generated in the mouse embryo. It is expressed by all HSCs (de Bruijn et al., 2002, Ma et al., 2002), about 30% of HPCs (Solaimani Kartalaei et al., 2015) and progenitors with lymphoid potential in the E10.5/E11.5 AGM (Li et al., 2014). To examine whether head HPCs express this marker, E10.5 and E11.5 whole head and subregions were isolated from Ly6aGFP embryos and sorted into GFP^+^ and GFP^−^ fractions. Of the 271 CFU-C obtained from the E10.5 whole head (Fig. 2A), majority (87%) were in the GFP^−^ fraction. Although few CFU-C were found in the GFP^+^ fraction, all colony types were found in both the GFP^+^ (except CFU-E) and GFP^−^ fractions. CFU-C localized to all three subregions and were in equal distribution when the total number of cells in each region was considered (see legend to Fig. 1A).

In the E11.5 whole head the total number of CFU-C increased by 9–12 fold (3287) as compared to E10.5 (Fig. 2B). The GFP^−^ fraction still contained most (77.2%) of the CFU-C (2538). However, the number of CFU-C in the GFP^+^ fraction increased by a factor of 10. Over 80% of the CFU-C, both GFP^+^ and GFP^−^, are localized to the FB and HBA regions. The GFP^+^ fractions (whole head and subregions) contained about 78.5% of the CFU-Mix, whereas the GFP^−^ fraction contained all head CFU-E, and most BFU-E and CFU-M+GM (Fig. 2C). The fact that such a large percentage of CFU-C are GFP^−^ is agreement with the data of others supporting the notion that yolk sac derived primitive progenitors and EMPs colonize the brain, and that these cells are Sca1 negative (McGrath et al., 2015). These head results are similar to AGM data showing that Ly6aGFP expression correlates to cells with more immature hematopoietic potential and suggest that changes in the hematopoietic cell composition could be related to *in situ* hematopoietic cell generation.

### 3.3 HSCs localize to HBA and FB, and are exclusively Ly6aGFP positive

**Fig. 3:**
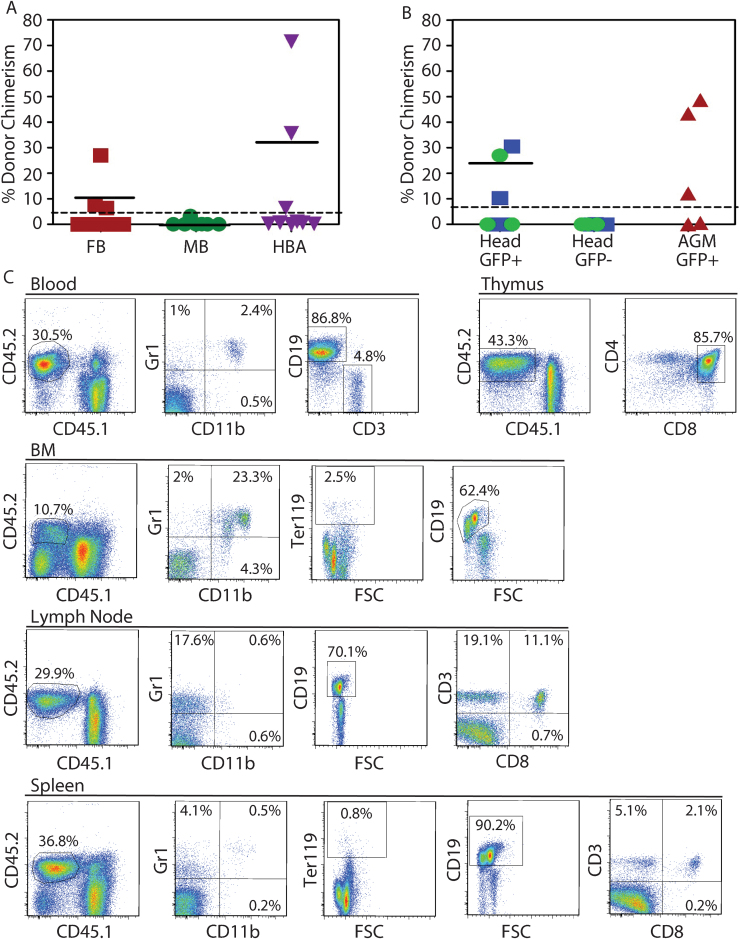
Head HSCs are GFP^+^ and localized to the HBA and FB. (A) Percentage donor cell chimerism in the peripheral blood of adult *Ly5.1* recipients injected with *Ly5.2* cells (4–7 embryo equivalents (ee)/recipient) from E11.5 forebrain (FB, red), midbrain (MB, green) and hindbrain with brachial arches (HBA, purple) at 16 weeks post-transplantation. n=3. Chimerism was analysed in peripheral blood by flow cytometry for CD45.2. (B) Hematopoietic repopulation by E11.5 (blue squares) and E12.5 (green dots) *Ly6aGFP* head cells. GFP^+^ and GFP^-^ sorted head cells were injected into irradiated adult *Ly5.1* recipients and percentage donor chimerism (Ly5.2) determined by flow cytometry at 16 weeks post-transplantation. 0.5–1.8 ee of E11.5 (n=5) and 0.5–2.5 ee of E12.5 (n=3) were injected per recipient. E11.5 AGM GFP^+^ cells were injected as a control (1.1–2.15 ee/recipient, n=5). Horizontal lines indicate mean chimerism of engrafted mice (only those mice with 5% or greater donor chimerism were considered engrafted. (C) Representative multilineage repopulation analysis of a head GFP^+^ recipient mouse (30.5% engrafted) in panel B. Flow cytometric analysis for percentage of donor-derived (CD45.2^+^) hematopoietic cells in peripheral blood, BM, thymus, lymph node and spleen is shown. Gated CD45.2^+^ cells were analysed for myeloid lineage cells (granulocytes, Gr1+ and macrophages, CD11b^+^), B cells (CD19^+^), T cells (CD4^+^/CD8^+^ or CD3^+^) and erythroblasts (Ter119^+^). FSC=forward scatter.

Previously, we demonstrated by the *in vivo* transplantation assay that HSCs are present in the E10.5 head at limiting numbers, and that head HSC numbers increase at E11.5 and E12.5 (Li et al., 2012). To examine whether HSCs are localized to any of the head subregions, we transplanted E11.5 FB, MB and HBA cells and measured donor cell hematopoietic engraftment at 16 weeks post-injection (Fig. 3A). The most robust and frequent engraftment was found with HBA cells, with 33% of recipients showing multilineage chimerism (36.5±32.8%). HSCs were found also in the FB, but at a lower frequency (25%) and yielded lower chimerism levels (13.6±11.6%). Only rare, very low engraftment (0.1%, 3.3%) was found from MB cells. Secondary transplantations performed with HBA-repopulated primary recipient BM (71.5% and 45.4% donor-derived), showed an average of 8.7±2.5% (n=3) and 14.9±4.6% (n=3) long-term donor-derived engraftment, thus demonstrating that HBA HSC are self-renewing.

To examine whether head HSCs are Ly6aGFP expressing, E11.5 and E12.5 GFP^+^ and GFP^−^ head cells were injected into irradiated adult recipients. At 16 weeks post-transplantation only GFP^+^ cells provided high level, multilineage chimerism (22.6±10.8%; Fig. 3C). These HSCs were self-renewing as determined by secondary transplantations of BM from primary (30.5% and 10.3%) repopulated recipients (secondary recipient engraftment was 17.8 (n=1) and 20.7% (n=1) respectively). The fact that all HSC from the head are Ly6aGFP-expressing verifies the robustness of this marker in the identification of all engrafting HSC independent of developmental stage or tissue localization. Although we did not sort GFP^+^ and GFP^−^ cells from the three subregions, the predominance of HSCs in the HBA is of interest. In this regard, the CFU-C data corroborate the HBA predominance of robust GFP^+^ hematopoietic cells that should include GFP^+^ HSCs.

### 3.4 Phenotypic head HPSCs do not appear in clusters

**Fig. 4:**
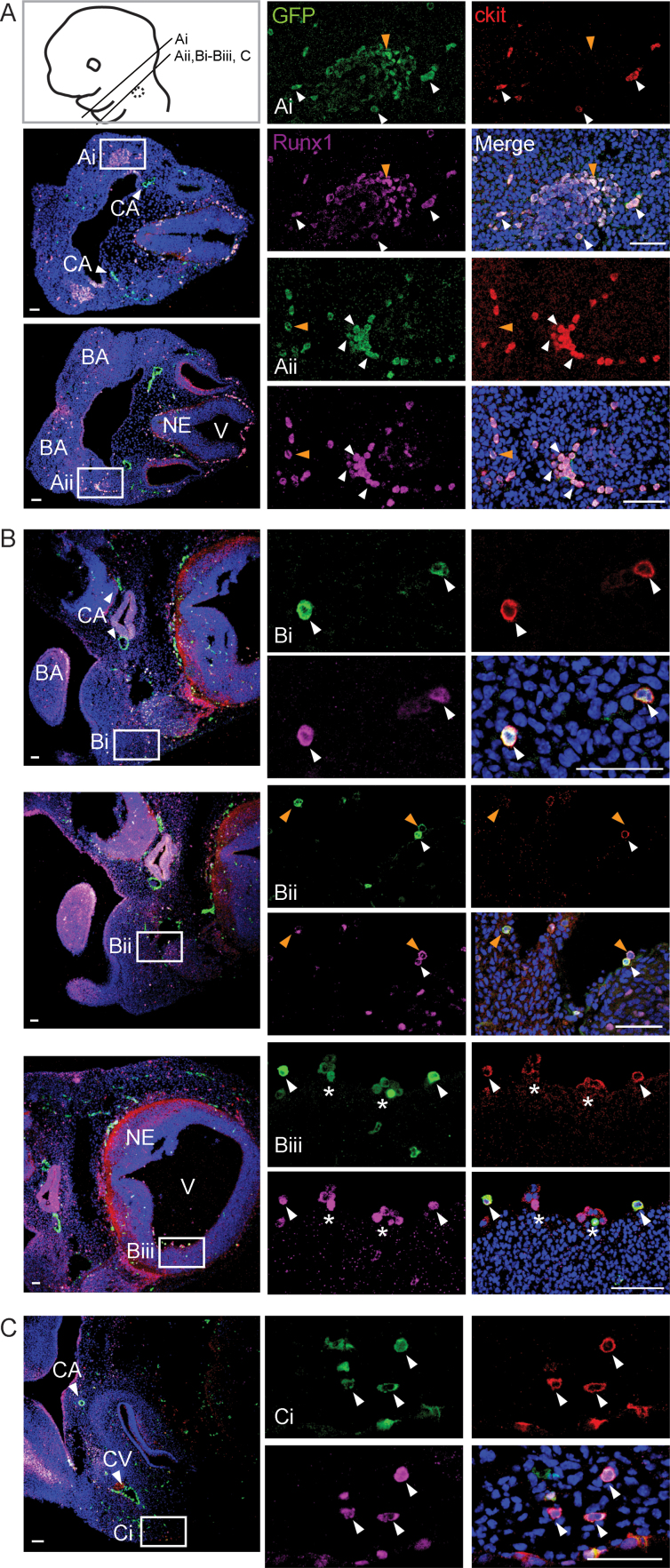
Phenotypic hematopoietic cells are not in clusters. (A) Schematic drawing of an E11.5 head with diagonal lines indicating the plane of transverse sections in the confocal fluorescent images. Confocal images of immunostained *Ly6aGFP* E10.5 (A, 33 somite pairs (sp)), E11.5 (B, 46 sp) and E12.5 (C) hind brain/branchial arch (HBA) head sections. Ly6aGFP (green), Runx1 (magenta), ckit (red), and DAPI (blue) fluorescent images are shown individually and/or merged. Enlarged images of the boxed areas (Ai, Aii, Bi, Bii, Biii and Ci) are shown for GFP^low^Runx1^+^ckit^+^ cells (white arrowheads) and GFP^low^Runx1^+^ckit^-^ cells (orange arrowheads) cells. Bar=50 µm. E10.5 (n=2 embryos; 33 and 34 sp); E11.5 (n=3 embryos; 43, 45, 46 sp); E12.5 (n=2 embryos). CA=carotid artery, BA=branchial arches, NE=neuroepithelium, V=ventricle, CV=cardinal vein.

To localize HP/SCs, *Ly6aGFP* head sections were immunostained with antibodies specific for GFP, Runx1 and ckit, and confocal imaging was performed. We focused on the HBA because the carotid arteries were GFP^high^ expressing (Fig. 1D) and HSC activity was found in this subregion. E10.5 (Fig. 4A), E11.5 (Fig. 4B) and E12.5 (Fig. 4C) HBA sections showed Ly6AGFP^high^ expressing cells along the wall of the carotid arteries (CA). These vascular endothelial cells did not express ckit or Runx1, and is in agreement with the findings of others (Iizuka et al., 2016). GFP^low^Runx1^+^ cells were found scattered through the branchial arches (BA) of E10.5 heads (Fig. 4A). In some areas (Fig. 4Ai) the majority of these cells were ckit^−^ (enlarged inset, orange arrowheads). In another section from the same embryo (more posterior), the majority of the GFP^low^ Runx1^+^ cells were cKit^+^ (Fig. 4Aii). Interestingly, some of the GFP^low^Runx1^+^ckit^+^ cells are clustered (enlarged inset, white arrowheads), and they appear to be outside the vessels (Fig. 4Aii). GFP^low^Runx1^+^ckit^+^ cells were also found surrounding the neuroepithelium (NE) and the roof of the hindbrain (Fig. 4A, low magnification panels).

By E11.5, the head is much larger. GFP^low^Runx1^+^ckit^+^ cells are observed as single cells near and within the vein lateral to the CA (Fig. 4Bi-ii, white arrowheads). Some GFP^+^Runx1^+^ckit^−^ cells are also found in this area (Fig. 4Bii, orange arrowheads). Interestingly, the ventricle of the hindbrain contains both single GFP^+^Runx1^+^ckit^+^ hematopoietic cells (Fig. 4Biii, white arrowheads) and cluster-like GFP^+^Runx1^+^ckit^+^ cells (Fig. 4Biii, asterisks), which are along the ventricle face of the neuroepithelial layer. In the E12.5 HBA, GFP^+^Runx1^+^ckit^+^cells are found in the ventricle cavity and tongue primordia, and single GFP^+^Runx1^+^ckit^+^ and GFP^+^Runx1^+^ckit^−^ cells are scattered throughout the branchial arches (data not shown). The region lateral to the GFP^+^ cardinal vein (CV) contains single GFP^+^Runx1^+^ckit^+^ cells (Fig. 4Ci).

The variety of cell types in the embryonic head confounds the identification and localization of the emerging cells of the hematopoietic system. No GFP^high^Runx1^+^ckit^+^ cells were found in the lumen or closely associated with the wall of the carotid artery. Despite the lack of arterial clusters, it is possible that the single phenotypic HP/SCs (expressing Ly6aGFP, Runx1 and ckit) scattered throughout the HBA sections are in capillary beds/sinusoidal areas or veins which are not visible in these images. Interestingly, we found hematopoietic cells clustered in an ablumenal location near the cardinal vein (Fig. 4Aii).

Although *SP-A Cre* directed *RosaSTOPLacZ* recombination showed LacZ expression in vascular cells (CD31^+^) in the E12.5 embryonic head (Li et al., 2012), it is as yet uncertain how these HSCs are generated. Similar to the aorta (de Bruijn et al., 2002), we found here that Ly6aGFP is strongly expressed in the carotid arteries within the HBA and marks functionally potent HPC and HSC. The fact that phenotypic HP/SCs stand alone, isolated and without a special association with vasculature suggests that HP/SC generation in the head occurs differently. EHT may occur infrequently in the head and cells may immediately home to other niches. The zebrafish embryo models such an alternative mode of HP/SC generation and EHT. In the production of hematopoietic cells, aortic endothelial cells bulge in an ablumenal direction (Kissa and Herbomel, 2010) into the subaortic space, rather than the lumenal direction as in the mouse aorta (Boisset et al., 2010). No hematopoietic clusters form and these cells move immediately to the circulation in the zebrafish axial vein (Kissa and Herbomel, 2010).

Others have shown that the early head-fold and neuroectodermal cells possess hemogenic potential if stimulated by morphogens (Baron, 2001, Dyer et al., 2001, Kanatsu and Nishikawa, 1996) and hence our result showing GFP^+^Runx1^+^ckit^+^ cells along the neuroepithelium is intriguing. Hence, the process of head HP/SC generation is likely to be different, or alternatively, EHT may be too infrequent/rapid to be observed. In this regard, it is of interest to further understand the microenvironment (morphogens, shear stress/pressure, metabolism, and oxygen levels) of the head and how it promotes hematopoietic cell development. Knowledge of what factors and the balance of the factors necessary for the efficient production of HPC and HSC from the head as compared to the aorta and other hemogenic tissues such as the yolk sac and placenta, should inform current approaches attempting HSC production *ex vivo*.

## Authorship contributions

ZL performed research. CSV and SAM performed/analysed flow cytometric data and CSV provided mouse support. ZL and ED designed experiments, analysed and interpreted data. ZL and ED wrote the manuscript.

## References

[bib1] Baron M. (2001). Induction of embryonic hematopoietic and endothelial stem/progenitor cells by hedgehog-mediated signals. Differentiation.

[bib2] Bertrand J.Y., Chi N.C., Santoso B., Teng S., Stainier D.Y., Traver D. (2010). Haematopoietic stem cells derive directly from aortic endothelium during development. Nature.

[bib3] Boisset J.C., van Cappellen W., Andrieu-Soler C., Galjart N., Dzierzak E., Robin C. (2010). In vivo imaging of haematopoietic cells emerging from the mouse aortic endothelium. Nature.

[bib4] de Bruijn M.F., Ma X., Robin C., Ottersbach K., Sanchez M.J., Dzierzak E. (2002). Hematopoietic stem cells localize to the endothelial cell layer in the midgestation mouse aorta. Immunity.

[bib5] Chen M.J., Yokomizo T., Zeigler B.M., Dzierzak E., Speck N.A. (2009). Runx1 is required for the endothelial to haematopoietic cell transition but not thereafter. Nature.

[bib6] Dyer M.A., Farrington S.M., Mohn D., Munday J.R., Baron M.H. (2001). Indian hedgehog activates hematopoiesis and vasculogenesis and can respecify prospective neurectodermal cell fate in the mouse embryo. Development.

[bib7] Dzierzak E., Speck N.A. (2008). Of lineage and legacy: the development of mammalian hematopoietic stem cells. Nat. Immunol..

[bib8] Frame J.M., Fegan K.H., Conway S.J., McGrath K.E., Palis J. (2016). Definitive hematopoiesis in the yolk sac emerges from Wnt-responsive hemogenic endothelium independently of circulation and arterial identity. Stem Cells.

[bib9] Ginhoux F., Greter M., Leboeuf M., Nandi S., See P., Gokhan S., Mehler M.F., Conway S.J., Ng L.G., Stanley E.R., Samokhvalov I.M., Merad M. (2010). Fate mapping analysis reveals that adult microglia derive from primitive macrophages. Science.

[bib10] Gomez Perdiguero E., Klapproth K., Schulz C., Busch K., Azzoni E., Crozet L., Garner H., Trouillet C., de Bruijn M.F., Geissmann F., Rodewald H.R. (2015). Tissue-resident macrophages originate from yolk-sac-derived erythro-myeloid progenitors. Nature.

[bib300] Iizuka, K., Yokomizo, T., Watanabe, N., Tanaka, Y., Osato, M., Takaku, T., Komatsu, N., 2016. Lack of phenotypical and morphological evidences of endothelial to hematopoietic transition in the murine embryonic head during hematopoietic stem cell emergence. PlosONE, pone.0156427 10.1371/journal.pone.0156427 PMC4882078 27227884

[bib11] Kanatsu M., Nishikawa S.I. (1996). In vitro analysis of epiblast tissue potency for hematopoietic cell differentiation. Development.

[bib12] Kissa K., Herbomel P. (2010). Blood stem cells emerge from aortic endothelium by a novel type of cell transition. Nature.

[bib13] Li Y., Esain V., Teng L., Xu J., Kwan W., Frost I.M., Yzaguirre A.D., Cai X., Cortes M., Maijenburg M.W., Tober J., Dzierzak E., Orkin S.H., Tan K., North T.E., Speck N.A. (2014). Inflammatory signaling regulates embryonic hematopoietic stem and progenitor cell production. Genes Dev..

[bib14] Li Z., Lan Y., He W., Chen D., Wang J., Zhou F., Wang Y., Sun H., Chen X., Xu C., Li S., Pang Y., Zhang G., Yang L., Zhu L., Fan M., Shang A., Ju Z., Luo L., Ding Y., Guo W., Yuan W., Yang X., Liu B. (2012). Mouse embryonic head as a site for hematopoietic stem cell development. Cell Stem Cell.

[bib15] Ling K.W., Ottersbach K., van Hamburg J.P., Oziemlak A., Tsai F.Y., Orkin S.H., Ploemacher R., Hendriks R.W., Dzierzak E. (2004). GATA-2 plays two functionally distinct roles during the ontogeny of hematopoietic stem cells. J. Exp. Med..

[bib16] Lux C.T., Yoshimoto M., McGrath K., Conway S.J., Palis J., Yoder M.C. (2008). All primitive and definitive hematopoietic progenitor cells emerging before E10 in the mouse embryo are products of the yolk sac. Blood.

[bib17] Ma X., de Bruijn M., Robin C., Peeters M., Kong A.S.J., de Wit T., Snoijs C., Dzierzak E. (2002). Expression of the Ly-6A (Sca-1) lacZ transgene in mouse haematopoietic stem cells and embryos. Br. J. Haematol..

[bib18] McGrath K.E., Frame J.M., Fegan K.H., Bowen J.R., Conway S.J., Catherman S.C., Kingsley P.D., Koniski A.D., Palis J. (2015). Distinct sources of hematopoietic progenitors emerge before HSCs and provide functional blood cells in the mammalian embryo. Cell Rep..

[bib19] Medvinsky A., Taoudi S., Mendes S., Dzierzak E. (2008). Analysis and manipulation of hematopoietic progenitor and stem cells from murine embryonic tissues. Curr. Protoc. Stem Cell Biol..

[bib20] North T.E., de Bruijn M.F., Stacy T., Talebian L., Lind E., Robin C., Binder M., Dzierzak E., Speck N.A. (2002). Runx1 expression marks long-term repopulating hematopoietic stem cells in the midgestation mouse embryo. Immunity.

[bib21] Ottersbach K., Dzierzak E. (2005). The murine placenta contains hematopoietic stem cells within the vascular labyrinth region. Dev. Cell.

[bib22] Rhodes K.E., Gekas C., Wang Y., Lux C.T., Francis C.S., Chan D.N., Conway S., Orkin S.H., Yoder M.C., Mikkola H.K. (2008). The emergence of hematopoietic stem cells is initiated in the placental vasculature in the absence of circulation. Cell Stem Cell.

[bib23] Robin C., Ottersbach K., Boisset J.C., Oziemlak A., Dzierzak E. (2011). CD41 is developmentally regulated and differentially expressed on mouse hematopoietic stem cells. Blood.

[bib24] Rybtsov S., Sobiesiak M., Taoudi S., Souilhol C., Senserrich J., Liakhovitskaia A., Ivanovs A., Frampton J., Zhao S., Medvinsky A. (2011). Hierarchical organization and early hematopoietic specification of the developing HSC lineage in the AGM region. J. Exp. Med..

[bib25] Solaimani Kartalaei P., Yamada-Inagawa T., Vink C.S., de Pater E., van der Linden R., Marks-Bluth J., van der Sloot A., van den Hout M., Yokomizo T., van Schaick-Solerno M.L., Delwel R., Pimanda J.E., van I.W.F., Dzierzak E. (2015). Whole-transcriptome analysis of endothelial to hematopoietic stem cell transition reveals a requirement for Gpr56 in HSC generation. J. Exp. Med..

[bib26] Yokomizo T., Dzierzak E. (2010). Three-dimensional cartography of hematopoietic clusters in the vasculature of whole mouse embryos. Development.

[bib27] Yokomizo T., Ng C.E., Osato M., Dzierzak E. (2011). Three-dimensional imaging of whole midgestation murine embryos shows an intravascular localization for all hematopoietic clusters. Blood.

[bib28] Zovein A.C., Hofmann J.J., Lynch M., French W.J., Turlo K.A., Yang Y., Becker M.S., Zanetta L., Dejana E., Gasson J.C., Tallquist M.D., Iruela-Arispe M.L. (2008). Fate tracing reveals the endothelial origin of hematopoietic stem cells. Cell Stem Cell.

